# New from old: recycling differentiated cells into regenerative cells using traditional Chinese medicine? A tribute to Professor Rongxiang Xu

**DOI:** 10.3389/fbioe.2024.1371707

**Published:** 2024-04-19

**Authors:** B. Péault

**Affiliations:** Department of Orthopaedic Surgery, Orthopaedic Hospital Research Center, UCLA, Los Angeles, CA, United States

**Keywords:** cell reprogramming, cell therapy, Chinese traditional medicine, skin regeneration, gut regeneration, stem cell, burn injury

## Introduction

New hopes in cell therapies have steadily arisen with ongoing progress in stem cell research, and innumerable conditions could be now treated and cured, from musculoskeletal injuries to diabetes to cardiac failure, to cite but a few, with the appropriate stem cells available. Arguably, the ideal therapeutic stem cells are those that naturally heal, repair, and replenish the target tissue in life, and research from the last decades has uncovered the presence of such committed, specialized stem cells in most organs. However, these cell lineage-specific regenerative cells are rare, difficult to identify and purify, and virtually impossible to culture for amplification as functionally intact, undifferentiated units. For this reason, and with the notable exception of hematopoietic stem cells, these are not presently amenable to clinical utilization.

## Dr Rongxiang Xu: a life commitment to organ regeneration

**FIGURE 1:**
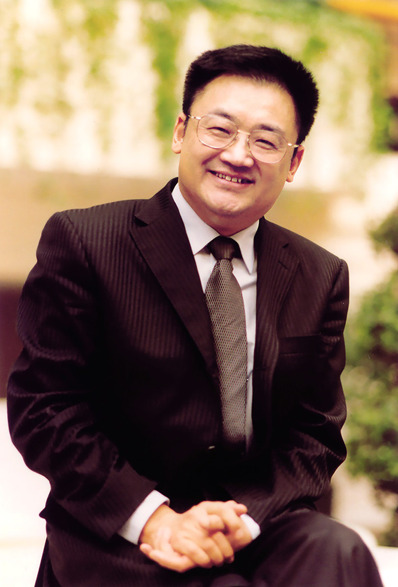
Professor Rongxiang Xu.

Born in 1958 to a poor rural family in China, Rongxiang Xu (Figure 1) was admitted to Qingdao Medical College in 1977 and eventually trained as a surgeon. From the earliest stages of his career, Dr. Xu was most concerned by the pain and distress endured by burn patients treated with conventional therapy. He dedicated himself to developing novel effective, less painful treatments of severe burns, even routinely inflicting burn injuries to his own skin to experiment new therapies. Dr. Xu and his research team finally developed the moist exposed burn therapy (MEBT) and moist exposed burn ointment (MEBO), new therapies utilizing the traditional Chinese pharmacopeia that contribute to restoring the normal structure and function of the skin and other tissues, thereby dramatically reducing pain, illness, and mortality. Dr Xu established the Guangming Chinese Medicine Burns Wounds and Ulcers Institute in Beijing in 1987. The same year, MEBT and MEBO were recognized as major achievements by the China National Scientific Committee and publicized nationwide by the China Ministry of Health in 1989. These treatments were recognized by the World Health Organization as most effective burn treatments in 2002. Dr. Xu has been granted dozens of international patents, as well as multiple awards including the Sixth Humanitarian Award by the American National Burn Victim Foundation, and European Golden Biatec International Award.

## Rongxiang Xu’s anticipated prediction of differentiated cell reprogramming

To interpret these remarkable achievements, Prof. Xu developed the theory that some of the pluripotent stem cells that build the embryo in early development also establish through adult life a minor subset of cells which, although seemingly terminally differentiated, retain strong multi-lineage developmental potential. He called these “potential regenerative cells” (PRCs), proposing these can be recruited into tissue repair and regeneration when no other progenitor cells are available (Xu et al., 2008). Although this hypothesis could not be fully tested experimentally at the time, it later turned out as strikingly visionary: on the one hand, it was demonstrated that mature cells can be reprogrammed into stem cells in culture, a discovery rewarded by attribution of the Nobel Prize in Medicine or Physiology in 2012 (Takahashi et al., 2007). Hence, transition from a functional, specialized cell back into a naïve, unbiased stem cell is, biologically, feasible and might explain the persistence of Rongxiang Xu’s PRCs in adult organs. Another example in support of PRC existence refers to mesenchymal stem cells. MSCs are multipotent cells that can differentiate in culture into bone, cartilage, fat, tendon, muscle, and indirectly support, via growth factor secretion, the regeneration of multiple other tissues (Pittenger et al., 2019). This extraordinary potential has stimulated the use of MSCs in over 2000 clinical trials in attempts to treat multiple conditions in cardiology, orthopaedics, cancerology, nephrology, and many other specialties including medical immunology, since MSCs are also immunosuppressive (see clinicaltrials.gov). Despite such a popularity, progress in MSC science has long been hindered by the unknown identity of native, tissue-resident mesenchymal stem cells, since these cells appear and expand in extended cultures of total, unselected cell populations dissociated from bone marrow, adipose tissue, umbilical cord, or other organs. Put in other words, MSCs are normally produced *in vitro* from elusive, rare ancestor cells that have long resisted description. This changed when multicolour, stringent flow cytometry cell sorting and sensitive differentiation assays were used for MSC prospective identification. In full support of the PRC concept proposed by Rongxiang Xu, innate tissue resident mesenchymal stem cells turned out to be differentiated perivascular cells of documented function. Pericytes, which enwrap capillaries and microvessels, regulate blood pressure and control angiogenesis. Equivalent cells populate the *tunica adventitia* at the periphery of larger blood vessels. Both purified pericytes and adventicytes give rise to *bona fide* mesenchymal stem cells when cultured *in vitro* (Crisan et al., 2008; Pittenger et al., 2019). The use of transgenic reporter mice, in which cell lineages can be tracked dynamically, has confirmed the progenitor cell potential of perivascular cells *in vivo* (Murray et al., 2017). In aggregate, all these results have confirmed that differentiated cells can be reprogrammed into regenerative cells in culture and in the living organism, thus supporting Dr Xu’s hypothesis on the existence of PRCs in adult organs.

## Harnessing traditional Chinese pharmacy to recruit potential regenerative cells: past attempts and recent achievements

Professor Rongxiang Xu contributed to the development of formulations, mostly inspired by traditional Chinese medicine, for regenerative cell (PRC) stimulation *in situ* and in culture. Such a supplement, for instance, named GIC, was claimed to regenerate the gastrointestinal mucosa, as well as nerves, in culture. More recently, the LifeRegen Inc. company developed a line of products inspired by Dr Xu’s research and patents were issued for a proprietary blend of natural ingredients produced *via* an original manufacturing process. All LifeRegen protocols rely on the infusion of combined black sesame oil, skullcap root, and beeswax. These products are considered GRAS (generally recognized as safe) by the FDA, and represent a scientific breakthrough merging ancient Chinese medicine recipes and Rongxiang Xu’s groundbreaking results that are, moreover, being supported and extended by ongoing current research. More precisely, *GI Balance* and *Juvenate Skincare* are LifeRegen’s proprietary formulations developed to nurture and renew the gut lining and create a cellular, rejuvenation promoting protective barrier for the skin, respectively.

## Conclusion

The insightful Prof. Rongxiang Xu predicted that some of the cells that constitute developed organs retain tissue healing potential, and hence can be qualified as “potentially regenerative cells”, and devised formulations believed to stimulate such cells into tissue regeneration. He left us a rich legacy of centers at prestigious institutions, such as the Rongxiang Xu Center for Regenerative Therapeutics at Beth Israel Deaconess Medical Center in Boston, the Rongxiang Xu Center for Regenerative Life Science at the University of Southern California, and the Rongxiang Xu College of Health and Human Services at Cal state LA.

The LifeRegen company has resumed, amplified, and diversified Dr Xu’s approach to regenerative medicine, and is now sponsoring clinical studies for the *GI Balance* supplement that in previous published research enabled regeneration and rejuvenation of the entire gut mucosa within 6 months. In a more fundamental investigation perspective, PRCs have been prospectively identified and characterized in depth, for instance as pericytes and other perivascular elements, and can be purified to homogeneity and in large numbers from multiple organs (Pittenger et al., 2019). This opens unforeseen possibilities in regenerative medicine research whereby such well-characterized potential regenerative cells can be selected from the bone marrow, pancreas, skin, adipose tissue and other organs and treated with natural supplements for enhanced proliferation, migration, and differentiation. Such protocols should be initialized in culture and further developed *in vivo* by transplantation into relevant animal hosts. In perspective are novel biomolecular manoeuvres to drive the improved healing and replenishment of, for instance but not exclusively, cardiovascular, musculoskeletal, and epithelial tissues.

After Rongxiang Xu passed away in 2015, his name was engraved on the celebrity wall at Harvard Medical School, while the latter permanently established the “Rongxiang Xu, MD., Professorship in Surgery in the Field of Regenerative Therapeutics”. Moreover, the state of California has named each last Tuesday in February as “Regenerative Medicine Day”, for public awareness and sustained respect to Dr. Rongxiang Xu. These posthumous tributes came to crown the outstanding life and career of a physician and researcher of exceptional commitment and scientific intuition, who gave a unique impetus to the nascent science of regenerative medicine and prophetically imagined some founding strategies thereof, while, simultaneously, exploiting centuries of Eastern medicine.
